# Suicide and Pesticide Use among Pesticide Applicators and Their Spouses in the Agricultural Health Study

**DOI:** 10.1289/ehp.1103413

**Published:** 2011-07-13

**Authors:** John D. Beard, David M. Umbach, Jane A. Hoppin, Marie Richards, Michael C. R. Alavanja, Aaron Blair, Dale P. Sandler, Freya Kamel

**Affiliations:** 1 Department of Health Science, College of Life Sciences, Brigham Young University, Provo, Utah, USA; 2 Biostatistics Branch, and; 3 Epidemiology Branch, National Institute of Environmental Health Sciences, National Institutes of Health, Department of Health and Human Services, Research Triangle Park, North Carolina, USA; 4 Westat, Durham, North Carolina, USA; 5 Occupational and Environmental Epidemiology Branch, Division of Cancer Epidemiology and Genetics, National Cancer Institute, National Institutes of Health, Department of Health and Human Services, Rockville, Maryland, USA

**Keywords:** farmers, pesticide applicators, pesticides, spouses, suicide

## Abstract

Background: An association may exist between pesticide exposure and suicide.

Objective: We sought to evaluate the existence of an association between pesticide use and suicide using data from the Agricultural Health Study (AHS), a prospective cohort study of licensed pesticide applicators and their spouses in Iowa and North Carolina.

Methods: Via linkage to state mortality files and the National Death Index, we identified 110 suicides occurring between enrollment in the AHS (from 1993 to 1997) and 31 May 2009, among 81,998 cohort members contributing 1,092,943 person-years of follow-up. The average length of follow-up was 13.3 years. AHS participants provided data on pesticide use and potential confounders via self-administered questionnaires at enrollment. We evaluated several measures of pesticide use: use of any pesticide, ever use of 50 specific pesticides, cumulative lifetime days of use and intensity-adjusted cumulative lifetime days of use of 22 specific pesticides, and ever use of 10 functional and chemical classes of pesticides. We used Cox proportional hazards regression models to estimate adjusted hazard ratios and 95% confidence intervals.

Results: After adjusting for age at enrollment, sex, number of children in family, frequency of alcohol consumption during the past 12 months, and smoking status, we found no association between prior pesticide use and suicide in applicators and their spouses. Results were the same for applicators and spouses together or for applicators alone and were consistent across several measures of pesticide use.

Conclusions: Our findings do not support an association between moderate pesticide use and suicide.

Several studies have reported higher suicide rates among farmers than the general population (Blair et al. 1993; Browning et al. 2008; Gunderson et al. 1993; Lee et al. 2002; Meltzer et al. 2008; Miller and Burns 2008; Page and Fragar 2002; Stallones 1990), although two studies found lower rates among Canadian farmers (Pickett et al. 1993, 1999). A review noted higher suicide rates among farmers than any other occupational group in the United Kingdom (Gregoire 2002).

Other studies suggested associations between chronic exposure to pesticides and suicide among farmers and other agricultural populations. In Australia, pesticide applicators had higher suicide rates than the general population (MacFarlane et al. 2009, 2010), whereas applicators in Italy had a lower rate of accidents and suicide (Torchio et al. 1994). Parrón et al. (1996) found higher suicide rates in an intensive agricultural area of southeastern Spain than in other areas with similar demographic and socioeconomic compositions; they tentatively attributed the increased rates to pesticide exposure. Colorado farmers potentially exposed to pesticides had higher suicide rates than the general population (Stallones 2006), and ecological and case studies suggested an association between organophosphate pesticide (OP) use and suicide (London et al. 2005).

The Agricultural Health Study (AHS) is a large, prospective cohort study of private pesticide applicators (mostly farmers), commercial applicators, and spouses of private applicators in Iowa and North Carolina. It was designed to study associations between cancer and other chronic diseases and farm-related exposures (Alavanja et al. 1996). Previously, AHS participants in the highest category of use of chlorpyrifos, an OP, were reported to be twice as likely to commit suicide as those who never used chlorpyrifos (Lee et al. 2007). Because that study was based on few cases and evaluated only one pesticide, we wanted to evaluate more fully the possible associations between the use of pesticides, particularly OPs, and suicide among applicators and their spouses in the AHS.

## Methods

*Population and case definition.* The AHS cohort, enrolled from 1993 to 1997, provided data on demographic and lifestyle factors, pesticide use, and other agricultural exposures at enrollment (Alavanja et al. 1996). Most private and commercial applicators were men (97% and 96%, respectively), whereas most spouses were women (99%). Mortality data including date and cause of death were obtained by linking the cohort to state mortality files and the National Death Index. We used the *International Classification of Diseases, 9th Revision* (ICD-9) [World Health Organization (WHO) 1977] codes to identify suicides from 1993 to 1998 and *10th Revision* (ICD-10) (WHO 1992) codes for those from 1999 to 2009. ICD-9 codes beginning with E95 or 95 or ICD-10 codes X60–X84 identified suicides listed as underlying or contributing causes of death. We excluded 129 individuals < 18 years of age at enrollment, 1 individual from a couple in which the private applicator and spouse had both committed suicide (the excluded individual was randomly chosen from the couple to avoid correlated death times), and an additional 7,528 cohort members missing covariate information. The analysis included a total of 81,988 cohort members: 48,098 private applicators [contributing 647,006 person-years (PY)], 4,781 commercial applicators (68,240 PY), and 29,119 spouses of private applicators (377,697 PY). The average length of follow-up was 13.3 years. We identified 110 suicides that occurred between enrollment and 31 May 2009.

The institutional review boards (IRBs) of the National Institutes of Health, Battelle Centers for Public Health Research and Evaluation (North Carolina field station), the University of Iowa (Iowa field station), and Westat (study coordinating center) approved this study, and the IRB of Brigham Young University exempted it. The study was explained to all potential participants, who indicated consent by returning the enrollment questionnaire.

*Exposure assessment.* Applicators and spouses provided information on pesticide use and other factors via questionnaires completed at enrollment (AHS 2011). This information included years of use (duration) and average days per year of use (frequency) for any pesticide in addition to ever use for 50 specific pesticides. For applicators, information was also collected on duration and frequency of use for 22 of the 50 pesticides. We evaluated suicide risk in relation to 10 pesticide categories (four functional: fumigants, fungicides, herbicides, and insecticides; and six chemical: phenoxy herbicides, triazine herbicides, carbamates, organochlorine insecticides, organophosphate insecticides, and pyrethroid insecticides) as well as individual pesticides and overall pesticide use.

For both applicators and spouses, we evaluated cumulative lifetime days of use of any pesticide. For applicators only, we also evaluated cumulative lifetime days of use and intensity-adjusted cumulative lifetime days of use for 22 individual pesticides, although we present results for only the 17 pesticides for which there were at least five exposed cases. Duration and frequency of use data were collected in seven and eight categories, respectively. Using values set to the midpoint of each category, or 50% greater than the lower bound in the highest category, we determined an applicator’s cumulative lifetime days of use for any pesticide and for each specific pesticide as the product of the duration and frequency values. We categorized cumulative lifetime days of use of any pesticide into quartiles based on the distribution of use for all applicators and spouses. We categorized specific pesticides into three levels based on the distribution of use for all applicators: *a*) none, *b*) used for less than or equal to the median lifetime days of use, and *c*) used for more than the median lifetime days. We also evaluated intensity-adjusted cumulative lifetime days of use, calculated as previously described (Dosemeci et al. 2002) and again categorized in three levels: none, ≤ median, > median.

*Statistical analysis.* We employed Cox proportional hazards regression models to estimate hazard ratios (HR) and 95% confidence intervals (CIs) for the association of suicide with each measure of pesticide use. Survival times were defined as the time (in days) between enrollment in the AHS and death for those who died from suicide (*n* = 110) or some other cause (*n* = 5,980) or the time between enrollment and 31 May 2009, for study participants still alive (*n* = 75,908). We ran models for applicators only and for applicators and spouses combined. There were too few suicides among spouses (*n* = 9) to analyze data on spouses alone. Possible confounding variables identified from prior reports (categorized as in Table 1) included age, sex, state of residence, race/ethnicity, education level, marital status, number of children in family (as a measure of social connection), size of farm worked last year, frequency of alcohol consumption during the past 12 months, smoking status, and ever diagnosed with heart disease or diabetes (as measures of chronic disease). We did not consider depression as a potential confounder, as it may be an intervening variable. Although spouses were not asked about number of children in family, farm size, or ever being diagnosed with heart disease, we inferred spouses’ answers for the first two items from applicators’ responses and based the last item on spouses’ responses to questions on myocardial infarction, angina, and arrhythmia.

**Table 1:** Characteristics of suicide cases and all pesticide applicators and spouses.

Cases*a*			Total*a*			Crude HR (95% CI)	Adjusted HR*b* (95% CI)
Characteristic	*n*	PY	*n*	PY			
Age at enrollment (years)												
18–35		27		164		17,741		245,354		1.14 (0.68, 1.91)		1.09 (0.65, 1.82)
36–45		32		243		24,406		331,257		Reference		Reference
46–65		36		217		33,448		442,276		0.84 (0.52, 1.35)		0.87 (0.54, 1.40)
> 65		15		79		6,403		74,056		2.07 (1.12, 3.82)		1.97 (1.07, 3.65)
Sex												
Male		100		648		51,679		698,643		Reference		Reference
Female		10		54		30,319		394,300		0.17 (0.09, 0.33)		0.18 (0.09, 0.34)
State of residence												
Iowa		61		406		55,734		743,676		Reference		Reference
North Carolina		49		296		26,264		349,267		1.72 (1.18, 2.50)		1.65(1.13, 2.41)
Applicator type or spouse												
Private applicator		89		569		48,098		647,006		Reference		Reference
Commercial applicator		12		81		4,781		68,240		1.30 (0.71, 2.37)		1.35 (0.73, 2.49)
Spouse (of private applicator)		9		51		29,119		377,697		0.17 (0.09, 0.34)		0.41 (0.07, 2.55)
Race/ethnicity												
White, non-Hispanic		102		669		79,567		1,060,879		Reference		Reference
Other		7		24		2,328		30,698		2.37 (1.10, 5.10)		2.15 (1.00, 4.63)
Education level												
≤ Some high school		10		53		5,737		73,003		1.39 (0.71, 2.76)		1.08 (0.54, 2.18)
High school graduate		48		324		36,854		489,551		Reference		Reference
GED, 1–3 years of vocational education beyond high school, or some college		37		224		22,292		299,743		1.26 (0.82, 1.94)		1.35 (0.87, 2.08)
≥ College graduate		13		87		15,725		212,110		0.63 (0.34, 1.16)		0.68 (0.37, 1.27)
Marital status												
Married or living as married		87		572		73,042		970,990		Reference		Reference
Divorced or separated		11		61		2,457		33,299		3.72 (1.99, 6.97)		2.70 (1.43, 5.09)
Widowed, never married, or other		12		69		6,420		87,645		1.54 (0.84, 2.82)		1.03 (0.54, 1.94)
Number of children in family												
≤ 1		44		265		19,642		266,403		Reference		Reference
> 1		66		437		62,356		826,540		0.48 (0.33, 0.70)		0.52 (0.34, 0.79)
Size of farm worked last year												
Did not work on a farm or < 5 acres		8		41		6,048		80,244		Reference		Reference
≥ 5 acres		81		553		69,263		922,473		0.88 (0.43, 1.82)		0.96 (0.46, 2.00)
Frequency of alcohol consumption during past 12 months												
Never		38		232		29,979		393,657		0.99 (0.67, 1.48)		1.11 (0.73, 1.68)
< Every day		67		435		51,239		689,157		Reference		Reference
Every day		5		35		780		10,129		5.04 (2.03, 12.51)		4.20 (1.69, 10.43)
Smoking status												
Never		39		275		49,202		658,739		Reference		Reference
Past		32		194		20,959		277,039		1.95 (1.22, 3.12)		1.57 (0.97, 2.54)
Current		39		234		11,837		157,164		4.20 (2.69, 6.55)		3.66 (2.34, 5.73)
Ever diagnosed with heart disease												
No		100		657		75,880		1,016,855		Reference		Reference
Yes		5		14		4,843		59,577		0.85 (0.34, 2.08)		0.87 (0.34, 2.19)
Ever diagnosed with diabetes (other than while pregnant)												
No		101		653		78,411		1,048,599		Reference		Reference
Yes		7		43		2,625		31,850		2.26 (1.05, 4.87)		2.25 (1.03, 4.91)
Ever diagnosed with depression												
No		85		586		76,603		1,022,481		Reference		Reference
Yes		18		85		4,059		53,221		4.06 (2.44, 6.75)		5.43 (3.25, 9.10)
**a**Information for specific covariates was missing for 0–8% of participants. **b**Adjusted for age at enrollment and sex.												

We evaluated all covariates significantly associated with suicide in both unadjusted and age- and sex-adjusted models as potential confounders (α = 0.05). We then performed manual backward selection using α = 0.05 to select a base model from the covariates identified in the first step. As an alternative model selection method, we performed manual forward selection using α = 0.05 and selected the same model as with the backward selection method. We evaluated whether additional covariates should be included in the base model with likelihood ratio tests (α = 0.05) and evaluated model fit using the Akaike information criterion and the Schwarz Bayesian criterion. The applicator-only base model included age at enrollment (18–35, 36–45, 46–65, > 65 years), number of children in family (≤ 1, > 1), frequency of alcohol consumption during the past 12 months (never, < every day, every day), and smoking status (never, past, current). The base model for applicators and spouses together also included sex.

Additional analyses included stratifying models by state of residence, number of children in family, and use of chemical-resistant gloves (applicators only). We formally compared HRs from the two strata using a 95% CI constructed from the standard error of the difference between HRs. To additional models, we added state, marital status, race/ethnicity, ever being diagnosed with diabetes, or cumulative lifetime days of use of any pesticide. We evaluated all four functional classes of pesticides together in a single model. Additionally, we evaluated carbamates, herbicides, organochlorine insecticides, OPs, and pyrethroid insecticides together in another model. Some analyses were repeated, restricting the sample to male applicators, applicators who personally used pesticides, individuals who did not report a physician diagnosis of depression, or cohort members ≤ 50 years of age at enrollment (because pesticide use among younger applicators is probably less likely to change during follow-up than use among older applicators). We separately evaluated suicides committed within 5 years of enrollment or more than 5 years past enrollment. We evaluated pesticide use during the year prior to enrollment. Finally, we used within-category medians to assess dose–response trends in cumulative lifetime days of use and intensity-adjusted cumulative lifetime days of use.

We used the P1REL090600 release of the Phase I data set, the REL090500.00 release of the demographic data set, and the REL201004.00 release of the mortality data set for this study and performed all analyses with SAS version 9.2 (SAS Institute Inc., Cary, NC).

## Results

In models adjusted for age at enrollment and sex, risk for suicide was significantly greater if participants *a*) were > 65 years of age compared with 36–45 years of age, *b*) were living in North Carolina, *c*) were of a race/ethnicity other than white/non-Hispanic, *d*) were divorced or separated compared with married or living as married, *e*) drank alcohol every day during the 12 months prior to enrollment compared with drank alcohol < every day, *f*) were current smokers compared with never smokers, or *g*) had ever been diagnosed with diabetes or depression (Table 1). Suicide risk was significantly lower for women than men and for participants with > 1 child compared with ≤ 1 child (Table 1).

We found little evidence that suicide was associated with overall pesticide use among applicators and spouses combined (Table 2). We saw no significant dose–response relationships between pesticide use (measured by duration, frequency, or cumulative lifetime days of use of any pesticide) and suicide. Only 37% of suicide cases (*n* = 41), all applicators, provided information on experiencing a high pesticide exposure event, and only seven cases experienced one (HR = 1.13; 95% CI: 0.50, 2.57). No cases reported being diagnosed with pesticide poisoning, but medical visits related to pesticide use were not significantly associated with suicide among applicators (Table 2).

**Table 2:** Suicide and pesticide use among applicators and spouses.

Cases*a*			Total*a*			Adjusted HR*b *(95% CI)
Variable	*n*	PY	*n*	PY		
Years personally mixed or applied pesticides										
None		7		54		13,904		180,992		Reference
≤ 5		29		152		13,009		175,839		1.53 (0.60, 3.87)
> 5		73		493		50,952		681,370		0.83 (0.33, 2.08)
										*p*_trend_ = 0.03
Days per year personally mixed or applied pesticides										
None		8		59		14,124		183,728		Reference
< 20		71		466		46,745		622,313		0.98 (0.42, 2.28)
≥ 20		30		175		16,997		232,178		0.89 (0.36, 2.20)
										*p*_trend_ = 0.67
Cumulative lifetime days personally mixed or applied pesticides*c*										
≤ 9		22		129		20,736		271,553		Reference
> 9–109		33		220		21,524		287,320		0.65 (0.37, 1.16)
> 109–370		26		182		19,747		265,712		0.51 (0.27, 0.94)
> 370		28		169		15,777		212,577		0.61 (0.33, 1.13)
										*p*_trend_ = 0.52
Medical visits related to pesticide use*d*										
No		91		592		49,022		663,393		Reference
Yes		9		57		3,492		47,305		1.32 (0.66, 2.62)
Functional pesticide classes*e* ever personally mixed or applied										
Fumigants										
No		85		542		69,238		920,494		Reference
Yes		25		160		12,178		164,901		0.98 (0.62, 1.54)
Fungicides										
No		74		470		61,607		818,170		Reference
Yes		36		232		19,761		266,561		0.89 (0.59, 1.34)
Herbicides										
No		14		83		20,326		265,005		Reference
Yes		96		619		61,406		824,414		0.69 (0.35, 1.36)
Insecticides										
No		18		112		22,312		291,593		Reference
Yes		92		590		59,560		799,710		0.85 (0.49, 1.49)
Chemical pesticide classes*f* ever personally mixed or applied										
Phenoxy herbicides										
No		37		219		25,338		336,166		Reference
Yes		67		455		40,178		542,779		0.70 (0.45, 1.06)
Triazine herbicides										
No		38		225		25,769		342,148		Reference
Yes		66		449		39,747		536,796		0.71 (0.47, 1.09)
Carbamates										
No		48		309		39,193		518,688		Reference
Yes		62		393		42,656		572,294		0.80 (0.54, 1.17)
Organochlorine insecticides										
No		66		445		54,499		726,683		Reference
Yes		44		257		26,745		356,697		0.88 (0.58, 1.35)
Organophosphate insecticides										
No		26		170		28,870		377,315		Reference
Yes		84		532		52,979		713,702		0.82 (0.50, 1.33)
Pyrethroid insecticides										
No		87		538		68,400		907,912		Reference
Yes		23		164		13,326		181,574		1.09 (0.68, 1.74)
Abbreviations: 2,4-D, (2,4-dichlorophenoxy)acetic acid; 2,4,5-T, (2,4,5-trichlorophenoxy)acetic acid; 2,4,5-TP, (*RS*)-2-(2,4,5-trichlorophenoxy)propionic acid; DDT, 1,1,1-trichloro-2,2-bis(4-chlorophenyl)ethane; DDVP, 2,2-dichlorovinyl dimethyl phosphate; EPTC, *S*-ethyl dipropyl(thiocarbamate). **a**Information for specific pesticide covariates was missing for 0–20% of participants. **b**Adjusted for age at enrollment, sex, number of children in family, frequency of alcohol consumption during the past 12 months, and smoking status. **c**Category boundaries set at the quartiles of the distribution of cumulative lifetime days of pesticide use for all applicators and spouses. **d**Not asked on the spouse questionnaire. Only results for applicators are shown. **e**Fumigants included aluminum phosphide, methyl bromide, carbon tetrachloride/carbon disulfide (80/20 mix), and ethylene dibromide. Fungicides included benomyl, captan, chlorothalonil, maneb/mancozeb, metalaxyl, and ziram. Herbicides included 2,4‑D; 2,4,5-T; 2,4,5-TP; alachlor; atrazine; butylate; chlorimuron ethyl; cyanazine; dicamba; EPTC; glyphosate; imazethapyr; metolachlor; metribuzin; paraquat; pendimethalin; petroleum oil; and trifluralin. Insecticides included aldicarb, aldrin, carbaryl, carbofuran, chlordane, chlorpyrifos, coumaphos, DDT, DDVP, diazinon, dieldrin, fonofos, heptachlor, lindane, malathion, parathion, permethrin (for animals), permethrin (for crops), phorate, terbufos, toxaphene, and trichlorfon. **f**Phenoxy herbicides included 2,4-D; 2,4,5-T; and 2,4,5-TP. Triazine herbicides included atrazine, cyanazine, and metribuzin. Carbamates included aldicarb, benomyl, carbaryl, and carbofuran. Organochlorine insecticides included aldrin, chlordane, DDT, dieldrin, heptachlor, lindane, and toxaphene. Organophosphate insecticides included chlorpyrifos, coumaphos, DDVP, diazinon, fonofos, malathion, parathion, phorate, terbufos, and trichlorfon. Pyrethroid insecticides included permethrin (for animals) and permethrin (for crops).										

For no specific functional or chemical class of pesticides, including OPs, was ever use significantly associated with suicide (Table 2). Only use of pyrethroid insecticides had an HR of > 1.0 (HR = 1.09; 95% CI: 0.68, 1.74), but that was not significant.

Among applicators and spouses together, ever use of individual pesticides was typically inversely associated with suicide, although estimates were often based on small numbers of exposed cases (Table 3). Five herbicides (atrazine, dicamba, imazethapyr, metolachlor, and pendimethalin) showed significant inverse associations; no positive association was statistically significant. The HR for (2,4,5-trichlorophenoxy)acetic acid (2,4,5-T), however, was elevated (HR = 1.55; 95% CI: 0.95, 2.53).

**Table 3:** Suicide and ever use of specific pesticides among applicators and spouses.

Cases*a*			Total*a*			Adjusted HR*b,c* (95% CI)
Ever personally mixed or applied*d*	*n*	PY	*n*	PY		
Fumigants										
Carbon tetrachloride/carbon disulfide (80/20 mix)		5		39		2,906		38,622		1.01 (0.41, 2.51)
Ethylene dibromide		5		30		1,851		25,051		1.32 (0.54, 3.27)
Methyl bromide		15		89		8,095		109,723		0.80 (0.46, 1.41)
Fungicides										
Benomyl		10		72		5,650		76,856		0.86 (0.45, 1.67)
Captan		8		44		5,805		76,640		0.87 (0.42, 1.81)
Chlorothalonil		13		83		4,698		63,921		1.32 (0.74, 2.37)
Maneb/mancozeb		7		48		5,330		72,448		0.64 (0.30, 1.40)
Metalaxyl		26		172		12,087		164,844		1.08 (0.68, 1.73)
Herbicides										
2,4-D		68		443		43,444		585,213		0.79 (0.52, 1.21)
2,4,5-T		24		194		10,539		140,942		1.55 (0.95, 2.53)
2,4,5-TP		10		90		4,703		63,527		1.25 (0.64, 2.44)
Alachlor		38		252		27,687		374,305		0.68 (0.44, 1.03)
Atrazine		56		402		37,529		506,172		0.64 (0.43, 0.96)
Butylate		26		231		16,164		220,059		0.91 (0.58, 1.45)
Chlorimuron ethyl		26		194		19,380		264,693		0.66 (0.42, 1.05)
Cyanazine		28		188		21,655		292,910		0.70 (0.45, 1.10)
Dicamba		33		271		26,363		356,274		0.63 (0.41, 0.98)
EPTC		17		148		10,678		145,053		0.97 (0.57, 1.66)
Glyphosate		77		509		49,703		667,712		0.94 (0.61, 1.45)
Imazethapyr		22		161		22,309		301,466		0.49 (0.30, 0.80)
Metolachlor		28		169		24,339		330,230		0.54 (0.35, 0.84)
Metribuzin		33		233		22,846		310,544		0.77 (0.50, 1.19)
Paraquat		19		152		12,658		172,340		0.70 (0.42, 1.16)
Pendimethalin		30		196		23,582		320,020		0.56 (0.36, 0.87)
Petroleum oil		32		211		24,022		324,356		0.65 (0.42, 1.00)
Trifluralin		41		290		27,759		375,427		0.79 (0.52, 1.19)
Insecticides										
Aldicarb		10		64		6,094		83,166		0.73 (0.37, 1.41)
Aldrin		11		57		9,298		123,387		0.71 (0.37, 1.38)
Carbaryl		55		357		37,073		497,220		0.99 (0.66, 1.48)
Carbofuran		23		181		13,691		184,258		0.99 (0.61, 1.59)
Chlordane		25		147		13,522		181,216		1.18 (0.73, 1.90)
Chlorpyrifos		40		263		22,784		308,881		0.99 (0.66, 1.48)
DDT		26		137		13,145		172,271		1.43 (0.85, 2.41)
DDVP		8		58		5,571		75,408		1.05 (0.51, 2.18)
Diazinon		34		208		18,850		254,163		1.16 (0.76, 1.76)
Fonofos		15		120		10,957		147,693		0.85 (0.49, 1.49)
Heptachlor		9		57		7,613		101,052		0.80 (0.39, 1.64)
Lindane		14		99		9,394		125,975		0.92 (0.52, 1.63)
Malathion		62		388		40,702		548,502		0.93 (0.61, 1.42)
Parathion		14		86		7,815		105,287		0.94 (0.53, 1.67)
Permethrin (for animals)		7		41		7,062		96,228		0.72 (0.33, 1.57)
Permethrin (for crops)		18		138		7,820		106,468		1.38 (0.82, 2.31)
Phorate		22		153		16,424		221,404		0.80 (0.49, 1.30)
Terbufos		24		176		19,527		263,680		0.67 (0.42, 1.07)
Toxaphene		9		64		7,092		94,595		0.72 (0.36, 1.45)
Abbreviations: 2,4-D, (2,4-dichlorophenoxy)acetic acid; 2,4,5-T, (2,4,5-trichlorophenoxy)acetic acid; 2,4,5-TP, (*RS*)-2-(2,4,5-trichlorophenoxy)propionic acid; DDT, 1,1,1-trichloro-2,2-bis(4-chlorophenyl)ethane; DDVP, 2,2-dichlorovinyl dimethyl phosphate; EPTC, *S*-ethyl dipropyl(thiocarbamate). **a**Information for specific pesticides was missing for 1–6% of participants. **b**Adjusted for age at enrollment, sex, number of children in family, frequency of alcohol consumption during the past 12 months, and smoking status. **c**For each pesticide, the applicators and spouses who did not use the pesticide served as the reference group. **d**Fewer than five cases used aluminum phosphide, coumaphos, dieldrin, trichlorfon, or ziram.										

Among applicators alone, none of the 22 individual pesticides showed a significant dose–response relationship between cumulative lifetime days of use and suicide (Table 4). Repeating those analyses with intensity-adjusted cumulative lifetime days of use, we found HRs that were generally < 1.0 but not significant (data not shown). We found significant inverse dose–response relationships between three intensity-adjusted pesticides and suicide: (2,4-dichlorophenoxy)acetic acid (2,4-D) (≤ 381 days vs. none: HR = 0.85; 95% CI: 0.53, 1.37; > 381 days vs. none: HR = 0.56; 95% CI: 0.33, 0.96; *p*_trend_ = 0.04), metolachlor (≤ 221 days vs. none: HR = 0.49; 95% CI: 0.26, 0.91; > 221 days vs. none: HR = 0.47; 95% CI: 0.25, 0.87; *p*_trend_ = 0.03), and terbufos (≤ 189 days vs. none: HR = 0.71; 95% CI: 0.38, 1.31; > 189 days vs. none: HR = 0.45; 95% CI: 0.22, 0.95; *p*_trend_ = 0.04).

**Table 4:** Suicide and dose response for specific pesticides among applicators.

Cumulative lifetime days of use of*c*	Cases*a*			Total*a*			Adjusted HR*b* (95% CI)
	*n*	PY	*n*	PY			
Fumigants										
Methyl bromide										
None		84		546		44,681		604,613		Reference
≤ 26*d*		9		59		3,919		53,240		0.98 (0.49, 1.98)
> 26		5		24		3,467		47,046		0.62 (0.25, 1.53)
										*p*_trend_ = 0.30
Fungicides										
Chlorothalonil										
None		89		577		47,968		648,713		Reference
≤ 28		6		29		2,041		27,769		1.32 (0.58, 3.03)
> 28		6		45		2,035		27,983		1.31 (0.57, 3.01)
										*p*_trend_ = 0.51
Herbicides										
2,4-D										
None		34		211		13,153		178,829		Reference
≤ 64		36		245		20,023		270,683		0.79 (0.49, 1.27)
> 64		25		165		18,453		249,665		0.61 (0.36, 1.04)
										*p*_trend_ = 0.11
Alachlor										
None		54		353		24,026		325,520		Reference
≤ 51		17		106		13,169		177,726		0.62 (0.36, 1.08)
> 51		18		118		12,351		168,066		0.69 (0.40, 1.18)
										*p*_trend_ = 0.24
Atrazine										
None		45		259		16,342		222,136		Reference
≤ 56		25		152		18,518		250,121		0.56 (0.34, 0.93)
> 56		27		219		17,104		231,261		0.68 (0.41, 1.10)
										*p*_trend_ = 0.37
Cyanazine										
None		64		402		29,498		399,275		Reference
≤ 39		15		84		10,993		147,671		0.74 (0.42, 1.30)
> 39		12		94		9,376		128,369		0.67 (0.36, 1.25)
										*p*_trend_ = 0.25
Dicamba										
None		61		343		24,905		337,385		Reference
≤ 39		14		133		13,824		186,574		0.49 (0.27, 0.88)
> 39		15		108		10,737		146,204		0.67 (0.37, 1.19)
										*p*_trend_ = 0.24
EPTC										
None		73		446		39,292		531,770		Reference
≤ 25		7		66		6,184		83,741		0.69 (0.32, 1.51)
> 25		9		75		3,797		52,139		1.36 (0.68, 2.74)
										*p*_trend_ = 0.36
Glyphosate										
None		24		136		12,692		171,041		Reference
≤ 39		42		276		21,334		288,296		1.04 (0.63, 1.72)
> 39		30		209		17,954		244,520		0.88 (0.51, 1.50)
										*p*_trend_ = 0.49
Cumulative lifetime days of use of*c*		Cases*a*				Total*a*				Adjusted HR*b* (95% CI)
		*n*		PY		*n*		PY		
Imazethapyr										
None		68		415		28,483		385,978		Reference
≤ 25		10		87		14,731		199,218		0.34 (0.17, 0.66)
> 25		12		74		6,219		84,531		0.87 (0.46, 1.62)
										*p*_trend_ = 0.75
Metolachlor										
None		65		437		26,995		364,519		Reference
≤ 51		10		29		12,185		164,929		0.37 (0.19, 0.73)
> 51		15		112		10,599		144,974		0.63 (0.36, 1.12)
										*p*_trend_ = 0.17
Trifluralin										
None		53		318		23,874		322,942		Reference
≤ 56		18		122		14,077		190,441		0.65 (0.38, 1.11)
> 56		22		163		11,453		155,823		0.98 (0.59, 1.63)
										*p*_trend_ = 0.80
Insecticides										
Carbofuran										
None		69		408		36,836		499,756		Reference
≤ 25		12		90		8,025		107,940		0.89 (0.48, 1.65)
> 25		10		84		4,665		63,161		1.19 (0.61, 2.32)
										*p*_trend_ = 0.59
Chlorpyrifos										
None		61		394		30,791		415,705		Reference
≤ 25		17		120		11,766		159,792		0.77 (0.45, 1.33)
> 25		20		124		9,443		128,517		1.12 (0.67, 1.86)
										*p*_trend_ = 0.54
Fonofos										
None		76		471		39,562		536,183		Reference
≤ 25		6		40		5,625		75,713		0.65 (0.28, 1.51)
> 25		8		71		4,498		61,030		1.05 (0.50, 2.18)
										*p*_trend_ = 0.88
Permethrin (for crops)										
None		75		469		42,318		572,345		Reference
≤ 25		9		71		4,441		60,458		1.17 (0.58, 2.34)
> 25		5		40		2,538		34,973		0.99 (0.40, 2.45)
										*p*_trend_ = 0.99
Terbufos										
None		67		423		31,348		424,772		Reference
≤ 39		12		76		9,353		125,951		0.68 (0.36, 1.26)
> 39		9		73		8,841		120,368		0.53 (0.26, 1.06)
										*p*_trend_ = 0.07
Abbreviations: 2,4-D, (2,4-dichlorophenoxy)acetic acid; EPTC, *S*-ethyl dipropyl(thiocarbamate). **a**Information for specific pesticides was missing for 0–4% of applicators. **b**Adjusted for age at enrollment, number of children in family, frequency of alcohol consumption during the past 12 months, and smoking status. **c**Fewer than five cases used captan, coumaphos, 2,2-dichlorovinyl dimethyl phosphate (DDVP), permethrin (for animals), and trichlorfon. **d**Category boundaries set at the median cumulative lifetime days of use of each pesticide among applicators who used them (i.e., both cases and controls).

When we stratified by state of residence or number of children in family separately and used the ever-use herbicide variables for applicators and spouses, we observed the same results in each stratum: generally inverse associations between herbicides and suicide with a few being significant (data not shown). Formal comparison of the stratum-specific HRs showed no significant differences. Data on use of chemical-resistant gloves were available only for applicators. Stratification by this variable yielded similar results in both strata for general use and herbicide variables (data not shown).

Results remained unchanged when state, marital status, race/ethnicity, ever being diagnosed with diabetes, or cumulative lifetime days of use of any pesticide were added to the models one at a time (data not shown). Evaluating all four functional pesticide classes together in a single model or evaluating carbamates, herbicides, organochlorine insecticides, OPs, and pyrethroid insecticides together in another model did not change results (data not shown). Excluding cohort members who had been diagnosed with depression changed results slightly: a few more pesticides were inversely associated with suicide and a few more inverse associations were significant (data not shown). Restricting analyses to male applicators, to applicators who personally used pesticides, or to cohort members ≤ 50 years of age at enrollment did not change results (data not shown). Likewise, evaluating pesticide use during the year prior to the enrollment of cohort members in the AHS did not change results (data not shown).

Evaluating suicides committed more than 5 years after enrollment in the AHS showed no significant associations between suicide and general pesticide use or the functional and chemical class pesticide variables, although most HRs were slightly more negative (data not shown). Similarly we found no significant associations between pesticide use and suicide committed within 5 years of enrollment (data not shown).

## Discussion

We found no association between pesticide use up to enrollment in the AHS and subsequent incidence of suicide in pesticide applicators and their spouses. This finding was consistent for use of any pesticide or individual pesticides, for functional or chemical classes, and for cumulative lifetime days of pesticide use. Results were the same for applicators and spouses together or for applicators only.

Although many studies have reported higher suicide rates among farmers and pesticide applicators than the general population (Blair et al. 1993; Browning et al. 2008; Gunderson et al. 1993; Lee et al. 2002; MacFarlane et al. 2009, 2010; Meltzer et al. 2008; Miller and Burns 2008; Page and Fragar 2002; Stallones 1990), others have found lower rates (Pickett et al. 1993, 1999; Torchio et al. 1994). A recent mortality analysis showed lower suicide rates among AHS participants than among the general populations of Iowa and North Carolina at least 15 years of age, although deaths due to unintentional injuries were elevated (Waggoner et al. 2011). The lower suicide rate in the AHS could reflect misclassification of suicides as unintentional injuries (e.g., collision with objects).

Another possible explanation for the lower suicide rate in the AHS is that individuals at risk of suicide may be less likely to enroll initially. This self-selection, if nondifferential by exposure, should not bias estimated HRs, although it might reduce precision. To reduce the influence of the healthy worker effect on their standardized mortality ratios (SMRs), Waggoner et al. (2011) calculated relative SMRs (rSMRs), a ratio of a cause-specific SMR and the SMR for all other causes except the cause of interest. The rSMR for suicide among applicators was 1.06 (95% CI: 0.87, 1.28) (Waggoner JK, personal communication), suggesting that the deficit in suicides observed in the AHS may be due to the healthy worker effect.

Previous studies that reported higher suicide rates among farmers and pesticide applicators had less-detailed pesticide exposure information than that available in the AHS (London et al. 2005; Parrón et al. 1996; Pickett et al. 1998; Stallones 2006). For example, one study inferred exposure from the fact that the study area had the highest density of greenhouses in the world (Parrón et al. 1996), and another inferred exposure from the occupation listed on death certificates (Stallones 2006). Pickett et al. (1998) found no association between suicide among Canadian farmers and three exposure measures: *a*) acres sprayed with herbicides, *b*) acres sprayed with insecticides, and *c*) the costs of purchased agricultural chemicals. We did not replicate a previous finding from the AHS that suggested an association of chlorpyrifos with suicide (Lee et al. 2007). We had 65% more suicide cases among applicators using chlorpyrifos (*n* = 38) than in the previous study (*n* = 23), suggesting that the present results may be more reliable.

Other rural lifestyle factors besides pesticide use may explain the higher suicide rates among farmers observed in other studies. Farmers have ready access to lethal means such as guns, pesticides, and other chemicals (Booth et al. 2000; Goldney 2002; Gregoire 2002; Hawton and van Heeringen 2009; Nock et al. 2008). The social change that occurs when small farms are incorporated into larger ones could also contribute to higher suicide rates via higher unemployment rates and a breakdown in family relationships (Goldney 2002; Stallones 1990).

Factors previously reported to increase suicide risk, such as old age, male sex, unmarried status, social isolation, frequent alcohol consumption, frequent smoking, and being diagnosed with depression, did so in our study also, suggesting that there is nothing unusual about suicide cases in the AHS. Further, AHS farmers are generally similar to other U.S. farmers (Lynch et al. 2005), so our results should be generalizable.

The tendency for herbicide use to be inversely associated with suicide, even if only a few associations were statistically significant, is perplexing. These inverse associations did not change meaningfully in a variety of analyses. Individuals who later committed suicide may have experienced mental problems, financial stress, stress in general, or other problems that might have caused them to farm less or use herbicides less than they otherwise would, leading to apparent inverse associations between herbicide use and suicide. However, the prospective design of our study helps alleviate this concern, particularly because results were similar for suicides committed within 5 years of enrollment and those committed later.

Our results may be surprising, given the association between pesticide use and physician-diagnosed depression found previously in the AHS (Beseler et al. 2006, 2008). Suicide, however, is not equivalent to depression. Only 17% (*n* = 18) of suicide cases in our study had been diagnosed with depression (Table 1). Other risk factors such as alcohol/substance use, mood disorders, and personality traits/disorders (e.g., impulsivity, aggressiveness, hopelessness, high emotional reactivity) are also important risk factors for suicide (Goldney 2002; Hawton and van Heeringen 2009; Nock et al. 2008) that cannot be ignored. Although depression may be underreported in the AHS, underreporting is not likely to account for our results, and our results did not change meaningfully when we excluded cohort members who had been diagnosed with depression.

Our study has several strengths. Selection bias was likely to be minimal, as 84% of eligible applicators and 75% of eligible spouses were enrolled in the AHS. The AHS collected detailed information on pesticide use before suicides occurred, including data on the use of individual pesticides and duration, frequency, and intensity of use. We were able to control for potential confounding factors. Finally, suicide from death certificates is a relatively valid outcome (Moyer et al. 1989).

Limitations include the small number of suicides (*n* = 110), which meant low power for estimating HRs for some individual pesticides. Further, participants were farmer owners/operators and their spouses or they were commercial applicators, that is, not farm workers. Therefore, results may not be generalizable to farm workers who may be more highly exposed than AHS farmers. Some information potentially useful for a suicide analysis was unavailable from AHS questionnaires. For example, information on financial situation or pertinent life events such as divorce or death of a family member was unavailable, as was information on personality or access to mental health care. Measures of acute high-intensity pesticide exposure were unavailable for most of the cohort, particularly suicide cases, and dose–response information was available for only 22 of 50 pesticides. Using data on past pesticide use instead of ongoing use could also misclassify pesticide use if ongoing use, and not past use, is what increases suicide risk.

In conclusion, we found little evidence that pesticide use increases suicide risk in pesticide applicators and their spouses. This finding, although based on relatively few suicides, was consistent across multiple measures of pesticide use and was robust to varying analytic strategies. Although this null finding needs confirmation in different populations, it could be reassuring to farming populations.
